# Identification of complex and cryptic chromosomal rearrangements by optical genome mapping

**DOI:** 10.1186/s13039-023-00636-2

**Published:** 2023-04-26

**Authors:** Shanshan Shi, Peizhi Huang, Ruiling Yan, Ruiman Li

**Affiliations:** 1 Fetal Medicine Center, Department of Obstetrics and Gynecology, The First Affiliated Hospital, Jinan University, No. 613 Huangpu West Road, Guangzhou, 510630 People’s Republic of China; 2 Prenatal Diagnosis Center, Department of Obstetrics and Gynecology, The First Affiliated Hospital, Sun Yat‐sen University, Guangzhou, People’s Republic of China

**Keywords:** Optical genome mapping, Complex chromosomal rearrangements, Cryptic translocation, Structural variation

## Abstract

**Background:**

Optical genome mapping (OGM) has developed into a highly promising method for detecting structural variants (SVs) in human genomes. Complex chromosomal rearrangements (CCRs) and cryptic translocations are rare events that are considered difficult to detect by routine cytogenetic methods. In this study, OGM was applied to delineate the precise chromosomal rearrangements in three cases with uncertain or unconfirmed CCRs detected by conventional karyotyping and one case with a cryptic translocation suggested by fetal chromosomal microarray analysis (CMA).

**Results:**

In the three cases with CCRs, OGM not only confirmed or revised the original karyotyping results but also refined the precise chromosomal structures. In the case with a suspected translocation not detected by karyotyping, OGM efficiently identified the cryptic translocation and defined the genomic breakpoints with relatively high accuracy.

**Conclusions:**

Our study confirmed OGM as a robust alternative approach to karyotyping for the detection of chromosomal structural rearrangements, including CCRs and cryptic translocations.

## Background

Genomic structural variations (SVs) usually refer to structural and quantitative chromosomal rearrangements that are longer than 100 bp [1] and have always been regarded as a direct or indirect cause of infertility, recurrent spontaneous abortion, birth defects and other events [2]. Unbalanced SVs refer to insertions, deletions, duplications, complex rearrangements and other events, while balanced SVs refer to copy-neutral variations with changes in the positioning or direction of the chromosome fragments but without gain or loss of chromosome fragments, such as balanced translocations and inversions [1, 3, 4]. Among the various SVs, complex chromosomal rearrangements (CCRs) and cryptic chromosomal rearrangements have received particular attention because they are difficult to identify with routine cytogenetic techniques [5–8].

CCRs describe SVs involving at least three breakpoints on two or more chromosomes, especially multiple translocations. Cryptic chromosomal rearrangements are characterized by chromosomal aberrations that are very subtle or located in the terminal zone and cannot be identified by conventional karyotyping, including cryptic translocations, inversions, insertions and other forms. The published data indicate that the unique and complex events involved in these rearrangements could be more complex than initially assumed [2, 9], but the underlying mechanisms remain elusive and need to be better characterized [10–12]. At this stage, some uncertainties in diagnosing complex/cryptic chromosomal rearrangements seem unavoidable.

Individuals with apparently balanced SVs are expected to be asymptomatic, but in fact, they are commonly at a high risk of infertility, recurrent spontaneous abortion, stillbirth, and delivering newborns with congenital malformations caused by chromosome unbalanced recombination events during the gametogenesis period [13]. Recent developments in high-throughput sequencing and chromosomal engineering technology have facilitated the accurate analysis of SVs in the human genome. In a diagnostic setup, diverse approaches have been adopted to detect SVs, including conventional karyotyping, fluorescence in situ hybridization (FISH), chromosomal microarray analysis (CMA), spectral karyotyping (SKY), and next-generation sequencing (NGS) [14, 15]. Moreover, optical genome mapping (OGM), which is independent of sequencing technology, has been applied to the detection of genomic SVs as a new clinical examination tool [16, 17]. The ability to detect all types of SVs in a single assay enabling an unbiased SV profile has shown unique advantages in hematological malignancies [18], solid tumors [19, 20], and prenatal disorders [17]. Thus, OGM offers marked advantages over conventional cytogenetics and standard molecular tests. This study applies OGM to perform high-precision analysis for CCRs and cryptic chromosomal rearrangements to address the complexity and occult nature of these rearrangements at the chromosomal and genomic levels and to investigate whether OGM could be used to improve clinical cytogenetics and provide more precise reproductive guidance.

## Methods

### Case presentation

A total of four patients who suffered from a history of adverse pregnancy or fertility, including two males and two females, were recruited to this study at the First Affiliated Hospital of Jinan University. These individuals had previously received peripheral blood chromosome karyotyping, which revealed that 3 of the patients carried unique CCRs and defined chromosomal break points at the microscopic level. The remaining patient was identified to have a normal karyotype, but a cryptic chromosomal rearrangement was highly suspected based on fetal CMA results. The patient demographic details are shown in Table 1. The patients signed informed consent forms before further testing was performed.

**Table 1:** Summarry of case information, original karyotypes and OGM results in 4 cases

Case	Age (years)	Clinical data	Original karyotype	Revised karyotype by OGM	CCRs or CR
1M	30	Infertility, Cryptozoospermia, Wife with early spontaneous miscarriage	46,X,Yqh + ,?del(21)(q21),add(22)(p13)	46,X,Yqh + ,t(3;21)(q27.1;q21.2),t(21;22)(q21.1;p11.2)	CCRs
2F	32	Infertility	46,XX,?ins(8;X)(q21.2;q22.2q22.1)	46,X,der(X)del(X)(q22.2q24),ins(10;X)(q26.12;q23q23), inv(8)(q21.2q24.3)	CCRs
3F	28	Early spontaneous miscarriages	46,XX,t(1;14)(p31;q21),ins(16;5)(q21;q15q13.1), der(5)inv(5)(q12q13.1)ins(16;5)(q21;q15q13.1)	46,XX,t(1;14)(p31;q21),ins(16;5)(q21;q15q12.3cth)	CCRs
4M	31	Fetus with unbalanced CNV	46,XY	46,XY,t(2;9)(q37.3;p24.1)	CR

*OGM* Optical genome mapping; *CCRs* complex chromosomal rearrangements; *CR* cryptic rearrangements; *cth* chromothripsis

### Conventional cytogenetic analysis

Routine G-banded karyotyping of peripheral blood lymphocytes from the 4 patients was performed using standard procedures. The metaphase chromosomes were analyzed according to the International System for Human Cytogenomic Nomenclature (ISCN) 2020 at 450-band resolution.

### CMA

In case 4, CMA was performed on the fetus (amniotic fluid) and the parents (peripheral blood) using a CytoScan 750 K chip (Affymetrix, Santa Clara, CA, USA) following the routine experimental procedure previously described [21]. The raw data were analyzed using Chromosome Analysis Suite (CHAS 4.0) software. Copy number variations (CNVs) were interpreted according to the American Institute of Medical Genetics and Genomics (ACMG).

### OGM

OGM was performed on the 4 cases. The brief experimental procedures were as follows, briefly: Ultra-high molecular weight (UHMW) gDNA was isolated from peripheral blood following the manufacturer's guidelines (Bionano Prep SP Frozen Human Blood DNA Isolation Protocol, Bionano Genomics, San Diego, CA, USA). Thereafter, the direct label and stain (DLS) technique was used according to the manufacturer’s instructions (Bionano Prep DLS Labeling Kit; Bionano Genomics, San Diego, CA) to label the DNA. The direct labeling enzyme 1 (DLE-1) reaction was carried out using 750 ng of high-molecular-weight gDNA to tag a specific 6-bp sequence (CTTAAG). Subsequently, the fluorescently labeled gDNA molecules were loaded on Saphyr chips for linearization and sequential imaging in massively parallel nanochannel arrays. De novo assembly and structural variant (SV) calling were performed via a de novo assembly pipeline through Bionano Access v1.2.1 (Bionano Genomics, San Diego, CA) and compared with Genome Reference Consortium GRCh38 (hg 38). Variant types such as insertions, deletions, inversion breakpoints, translocation breakpoints, and CNVs were detected with this pipeline [22, 23].

## Results

### Cases with CCRs

Our study used the OGM technique to reveal the refined genomic structures of three individuals with CCRs originally identified by conventional karyotyping.

#### Case 1

**Fig. 1:**
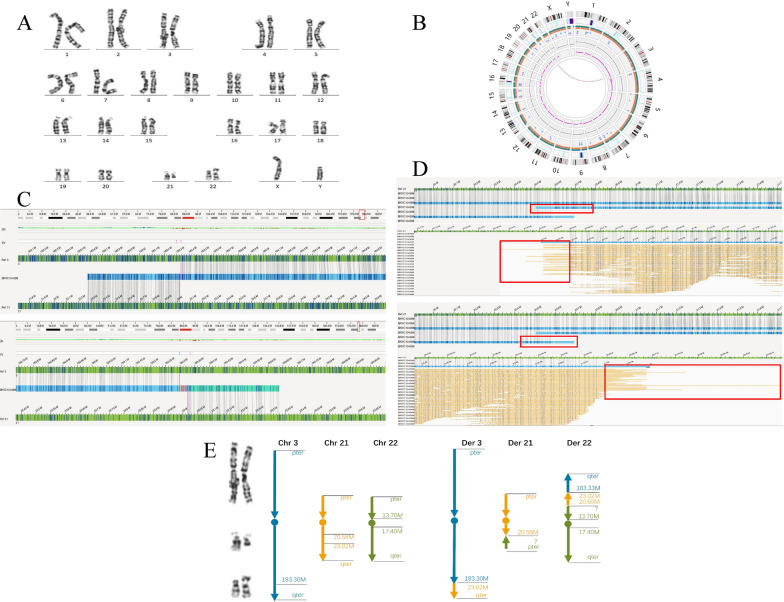
G-banding karyotyping and OGM results for case 1. **A** Karyotype showing a 46,X,Yqh+ ,?del(21)(q21),add(22)(p13). **B** Circos plot from OGM indicating a translocation event involving chromosomes 3 and 21. **C** OGM indicating a translocation event between chromosome 3q27.1 and chromosome 21q21.2. **D** The red boxes show suggesting unlabeled regions at chromosome 21q21.1 in OGM. Based on the combination of OGM and karyotype results, another balanced translocation was speculated: 46,XY,t(21;22)(q21.1;p11.2). **E** Schematic representation of the complex rearrangements involving chromosomes 3, 21 and 22. The complex chromosomal rearrangements were defined as 46,X,Yqh+ ,t(3;21)(q27.1;q21.2),t(21;22)(q21.1;p11.2)

A 30-year-old man was diagnosed with infertility and cryptozoospermia, and his wife had a history of miscarriage at 8 gestational weeks. Karyotyping of his peripheral blood indicated a karyotype of 46,X,Yqh+ ,?del(21)(q21),add(22)(p13), suggestive of a large deletion ranging from chromosome 21q21 to the terminus of the long arm and attachment of a large unknown chromosome segment to chromosome 22p13 (Fig. 1A). Nevertheless, the precise chromosome breakpoints and origins of these chromosomal fragments were not clearly defined.

Unexpectedly, further delineation of the chromosomes using OGM identified a breakpoint on chromosome 3q27.1, two breakpoints on chromosome 21 at q21.1 and q21.2 (divided into two segments with approximately 23.7 Mb and 2.4 Mb in size, respectively), and an assumed breakpoint on chromosome 22p11.2 (Fig. 1E). A clear reciprocal translocation between chromosomes 3q27.1 and 21q21.2 occurred without the generation of any fusion genes (Fig. 1B). The segment on chromosome 21 distal to 21q21.2 (~ 23.7 Mb) was translocated onto chromosome 3 at band q27.1 (Fig. 1C), but based on karyotype morphological features, the segment on chromosome 3 distal to 3q27.1 (~ 15 Mb) was translocated onto the chromosome 22p region instead of the chromosome 21q region (Fig. 1A). In addition, a small fragment (~ 2.4 Mb in size) resulting from another breakpoint on chromosome 21q21.1 was also considered to have translocated to the 22p11.2 region involving unlabeled regions (such as centromeres, telomere fragments and heterochromatin) [22], which might not be well defined by the OGM technique (Fig. 1D). Specifically, two translocation events were speculated in this case: first, one reciprocal translocation between chromosomes 3 and 21 with two breakpoints at 3q27.1 (at ~ 183.30 Mb) and 21q21.2 (at ~ 23.02 Mb), and second, a translocation between chromosome 21q21.1 (at ~ 20.58 Mb) and the 22p11.2 region, which was surmised based on karyotype morphological features. Overall, based on the combination of OGM and karyotyping results, the CCR was defined as 46,X,Yqh + ,t(3;21)(q27.1;q21.2),t(21;22)(q21.1;p11.2), as shown in Fig. 1.

#### Case 2

**Fig. 2:**
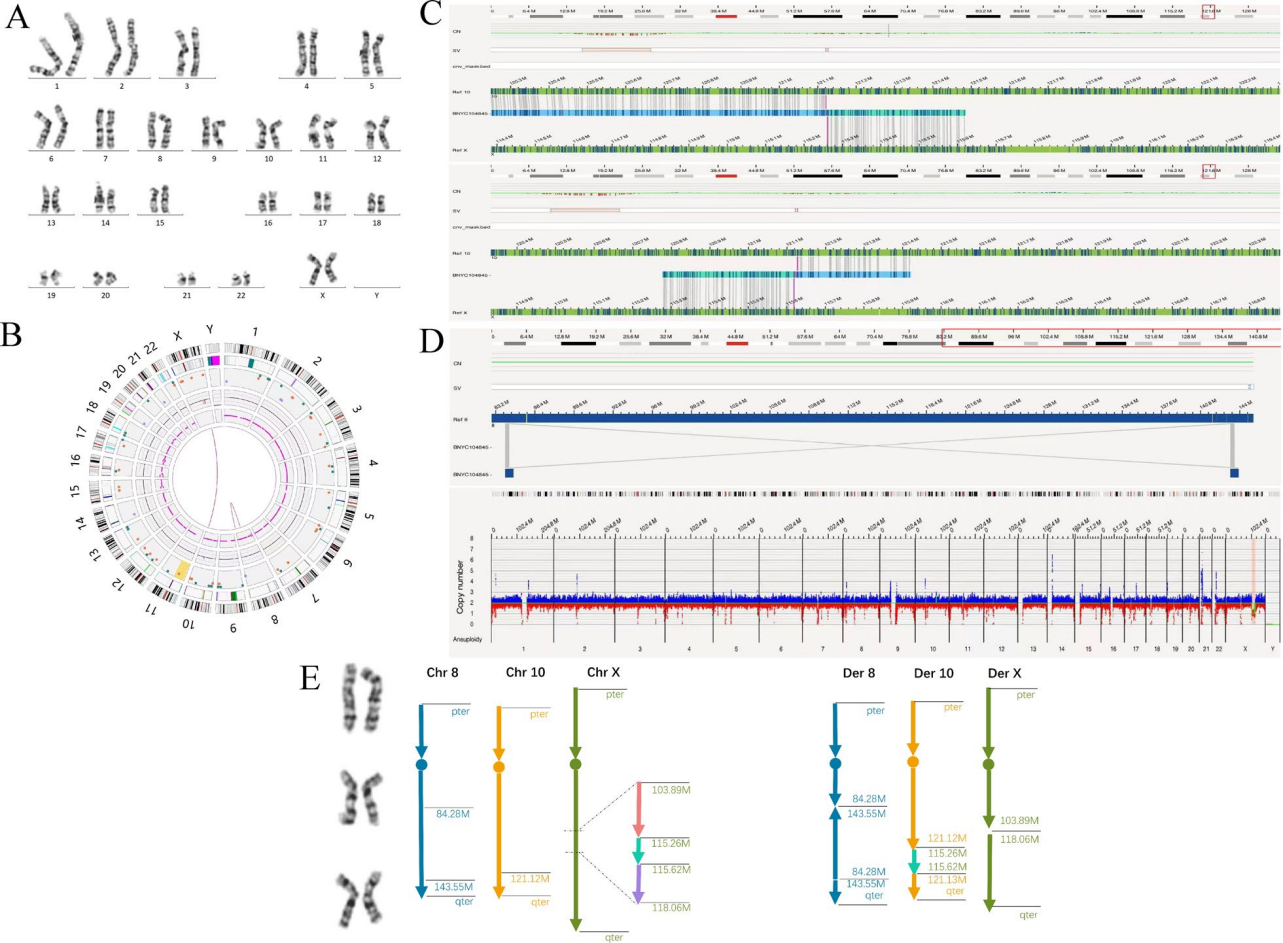
G-banding karyotyping and OGM results for case 2. **A** Karyotype revealing a 46,XX,?ins(8;X)(q21.2;q22.2q22.1). **B** Circos plot from OGM indicating a translocation event involving chromosome 10 and X, and an inversion event occurred on chromosome 8. **C** OGM indicating a cryptic insertion of Xq23q23 into 10q26.12. **D** Upper panel: OGM identifying a paracentric inversion event occurred between chromosome 8q21.2 and q24.3. Lower panel: OGM CNV plot showing two deletions on chromosomes Xq22.2q23 and Xq23q24. (E) Schematic representation of the complex rearrangements involving chromosomes 8, 10 and X. Altogether, the complex chromosomal rearrangements were defined as 46,X,der(X)del(X)(q22.2q24),ins(10;X)(q26.12;q23q23),inv(8)(q21.2q24.3)

A 32-year-old woman who suffered from infertility for 3 years was suspected to have a karyotype of 46,XX,?ins(8;X)(q21.2;q22.2q22.1) on the basis of initial karyotyping (Fig. 2A). It was suspected that one chromosome fragment from chromosome Xq22.2q22.1 was inserted into chromosome 8q21.2, presumably generating 3 chromosome breakpoints presumed between chromosomes 8 and X.

However, additional cryptic rearrangement events completely independent of the initial karyotype were identified by OGM (Fig. 2B). Chromosome X was detected to have a deletion involving the q22.2q24 region (~ 14.17 Mb). Nevertheless, the Xq22.2q24 region was broken into three subsegments and rearranged at the insertion site. The intermediate cryptic subsegment Xq23q23 [chrX:g.115,261,136–115,618,502] (~ 0.36 Mb) was inserted in a forward direction into chromosome 10q26.12 instead of chromosome 8 (Fig. 2C), while the other two subsegments, including Xq22.2q23 [chrX:103,893,393–115,261,136] (~ 11.37 Mb) and Xq23q24 [chrX:115,618,502–118,064,562] (~ 2.45 Mb) were deleted (Fig. 2D). Additionally, chromosome 8 was found to have a paracentric inversion spanning 8q21.2 to 8q24.3 (~ 59.27 Mb) (Fig. 2D). Details are shown in Fig. 2 and Table 2. In sum, the exact karyotype was 46,X,der(X)del(X)(q22.2q24),ins(10;X)(q26.12;q23q23),inv(8)(q21.2q24.3), which is unbalanced with partial monosomy for Xq.

**Table 2:** OGM refining chromosomal rearrangement structures in 2 cases with simplex insertion detected by karyotyping

Case	Der(acceptor)	Der(donor)	Deleted subsegments	Insertion site (acceptor)	Inserted segment (donor)
2F	ogm[GRCh38]der(10){10pter → 10q26.12( +)[121,121,448]::Xq23( +)[115,261,136] → Xq23( +)[115,618,502]::10q26.12( +)[121,127,008] → 10qter}	ogm[GRCh38]der(X){Xpter → Xq22.2( +)[103,893,393]::Xq24( +)[118,064,562] → Xqter}	ogm[GRCh38]del(X)(q22.2q23)chrX:g.103,893,393_115,261,136del, del(X)(q23q24)chrX:g.115,618,502_118,064,562del	K:8q21.2 O:10q26.12	K:Xq22.2q22.1 O:Xq23q23
3F	ogm[GRCh38]der(16){16pter → 16q21( +)[58,071,355]::5q15(-)[94,414,513] → 5q14(−)[77,885,855]::5q15(−)[97,742,767] → 5q15(−)[94,414,513]::5q15(−)[97,859,297] → 5q15(−)[97,752,968]::5q12.3( +)[64,373,122] → 5q13.3( +)[77,231,399]::16q21( +)[58,074,067] → 16qter}	ogm[GRCh38]der(5){5pter → 5q12.3( +)[64,373,122]::5q15( +)[97,859,297] → 5qter}	ogm[GRCh38]del(5)(q15q15)chr5:g.77,176,413_77,902,580del	K:16q21 O:16q21	K:5q15q13.1 O:5q15q12.3

*Der* Derivative chromosome; *K* karyotyping; *O* Optical genome mapping

#### Case 3

**Fig. 3:**
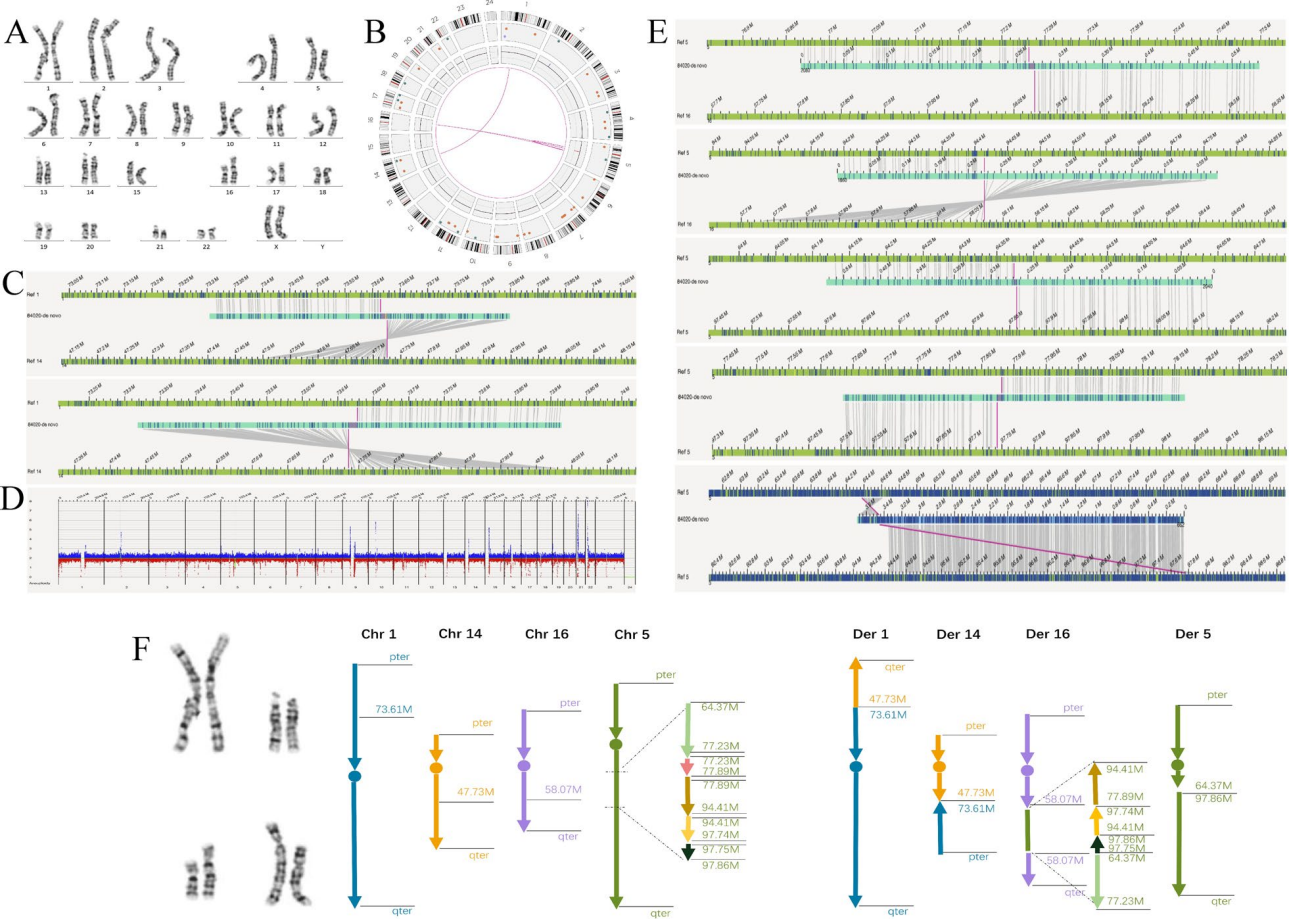
G-banding karyotyping and OGM results for case 3. **A** Karyotype revealing apparently balanced complex chromosomal rearrangement ascertained as 46,XX,t(1;14)(p31;q21),ins(16;5)(q21;q15q13.1),der(5)inv(5)(q12q13.1)ins(16;5)(q21;q15q13.1). **B** Circos plot from OGM indicating complex chromosomal rearrangements involving chromosomes 1, 5, 14, and 16. **C** OGM indicating a translocation event involving chromosomes 1 and 14. **D** OGM CNV plot showing a small deletion (0.7 Mb) on chromosome 5q14.1 (chr5:77,176,413–77,902,580). **E** OGM identifying complex chromosomal rearrangements involving chromosomes 5 and 16. **F** Ideograms of the derivative chromosomes are shown on the right and in different colors to illustrate the interchanges. Note that four of the five subsegments of chromosome 5q15q12.3 (light green, yellow, brown, black arrows) were rearranged and inserted into the long arm of chromosome 16q21, and a small deleted subsegment was not included in the insertion (pink arrow)

A 28-year-old female presented with fertility problems (early spontaneous miscarriages). Cytogenetic investigation revealed an apparently balanced karyotype of 46,XX,t(1;14)(p31;q21),ins(16;5)(q21;q15q13.1),der(5)inv(5)(q12q13.1)ins(16;5)(q21;q15q13.1). The complex rearrangement was found to have an apparent translocation between chromosomes 1p31 and 14q21, an insertion of chromosome 5q15q13.1 material within the 16q21 region, while the derivative chromosome 5 generated by the insertion exhibited more than one rearrangement, involving a paracentric inversion with the breakpoints at 5q12 and 5q13.1. This complex rearrangement was predicted to involve 4 chromosomes and 7 breakpoints (Fig. 3A).

Using OGM, we identified the rearrangements and revised the previously designated karyotype (Fig. 3B). In addition to the translocation between chromosomes 1p31 and 14q21, additional cryptic rearrangements in the chromosome 5q15q12.3 segment (chr5:64,370,000–97,860,000, ~ 33.49 Mb) were identified (Fig. 3E). The chromosome 5q15q12.3 segment was fragmented into 5 subsegments (indicating the occurrence of chromothripsis), of which 4 subsegments clustered at the insertion site of chromosomal 16q21 with complete rearrangement of orientation and position. In addition, 1 cryptic subsegment (chr5:77,176,413–77,902,580, ~ 0.7 Mb) was deleted but not inserted into chromosome 16q21 (Fig. 3D). Details are shown in Fig. 3 and Table 2.

### Case with cryptic chromosomal rearrangement

#### Case 4

**Fig. 4:**
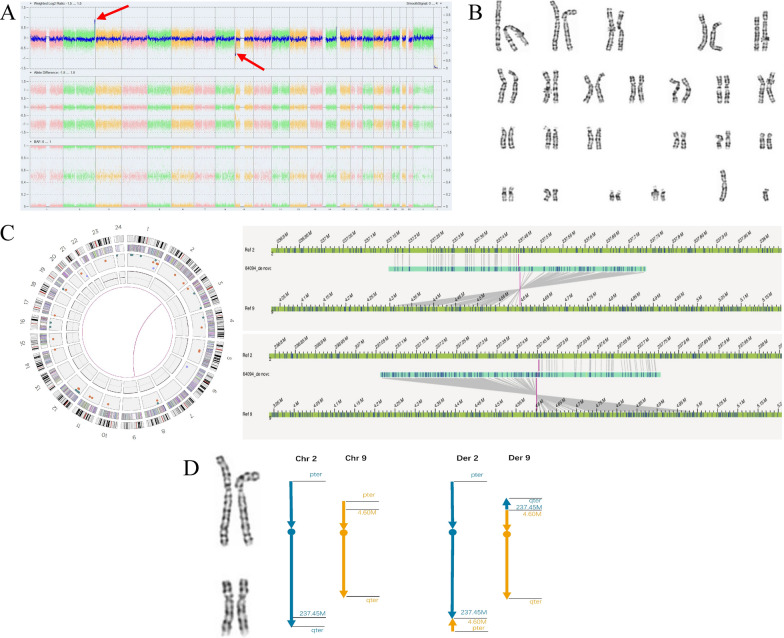
G-banding karyotyping, CMA and OGM results for case 4. **A** CMA identifying a 4.37-Mb terminal duplication of chromosome 2q37.3 (arr[GRCh38] 2q37.3(237,460,639–241,840,106) × 3) and a 4.39-Mb terminal deletion of chromosome 9p24.3p24.1 (arr[GRCh38] 9p24.3p24.1(208,455–4,600,613) × 1) in the fetus. **B** Karyotype showing a normal karyotype in the father of the fetus. **C** OGM indicating a cryptic translocation event between chromosomes 2 and 9 in the father of the fetus. **D** Schematic representation of the cryptic translocation between chromosomes 2q27.3 and 9p24.1 in the father of the fetus

A pregnant 27-year-old woman presented to genetic counseling for abnormal noninvasive prenatal testing (NIPT) results, suggesting a high risk of deletion at chromosome 9p. Karyotyping and CMA analysis of amniotic fluid cells were routinely performed. CMA detected a 4.37-Mb terminal duplication of chromosome 2q37.3 (arr[GRCh38] 2q37.3(237,460,639–241,840,106) × 3) and a 4.39-Mb terminal deletion of chromosome 9p24.3p24.1 (arr[GRCh38] 9p24.3p24.1(208,455–4,600,613) × 1) in the fetus (Fig. 4A), whereas karyotyping showed a normal karyotype. According to the ACMG CNV classification guidelines, deletion of 9p24.3p24.1 was defined as a likely pathogenic CNV, while duplication of 2q37.3 was defined as a CNV of uncertain significance (CNV-VUS). After detailed genetic counseling, the couples chose to terminate the pregnancy. The couples received further karyotyping and CMA tests of peripheral blood samples, and the test results were normal (Fig. 4B).

Therefore, a parental balanced translocation between chromosomes 2 and 9 was strongly suspected in this case based on fetal CMA results from amniocytes, but direct genetic evidence was lacking. Regrettably, FISH could not be performed because fluorescent probes for the two regions could not be easily or rapidly obtained clinically in our unit. Therefore, to further elucidate the balanced translocation and to determine the translocation breakpoints more precisely, OGM was carried out in the parents. The OGM indicated no chromosome SVs or CNVs were identified in the mother. However, a reciprocal translocation between chromosome 2 and chromosome 9 was identified in the father. The breakpoints were identified and narrowed down to approximately chr2:237,448,583 and chr9:4,600,788 (Fig. 4C and D).

## Discussion

Our study presents a case series of CCRs and cryptic chromosomal rearrangements. We fully resolved each rearrangement structure by combining the results of conventional karyotyping, CMA and OGM.

CCRs are rare structural rearrangements that involve more than two breakpoints with exchanges of chromosomal segments. The exact compositions and structures of CCRs are difficult to ascertain by karyotyping, CMA or short-read sequencing, resulting in difficulty in assessing the exact risk of unbalanced chromosomal changes in the offspring of the patients. Recent investigations have shown that CCRs are both more complex and more common than initially appreciated [24–26]. Various classifications of CCRs, such as three-way rearrangements, exceptional CCRs and double two-way translocations, have been proposed according to their structure, mode of transmission, number of chromosome breaks, and the involvement of intrachromosomal rearrangements (insertions, inversions, duplications) [27, 28]. Accordingly, three of the cases in our study would be classified as exceptional CCRs. Conventional karyotyping could be insufficient to define the fine structure of CCRs.

Insertional translocations (ITs) are rare events that require at least three breaks (two on the donor chromosome and one at the insertion site on the acceptor chromosome) and thus can be defined as CCRs. A recent study utilizing mate-pair genome sequencing (GS) revised previously designated karyotypes in 75.0% of the cases and identified additional cryptic rearrangements in 68.8% of the cases [9]. Similarly, in this study, we applied OGM to identify additional cryptic insertion rearrangements in two cases (Table 2). What these CCRs have in common is that the insertion segment of the donor chromosome was fragmented into two or more subsegments and was rearranged at the insertion site, and one or more cryptic subsegments of the insertion were not inserted into the acceptor chromosome. Consequently, these CCRs also involved copy number losses of subsegments of the donor chromosomes. This finding indicates that the most apparently simple CCRs (including insertions) can in reality often be more complex.

In case 1, a CCR involving three chromosomes (3, 21 and 22) without genomic gain and deletion was discovered. The refined karyotype was 46,X,Yqh+ ,t(3;21)(q27.1;q21.2),t(21;22)(q21.1;p11.2). Although the translocated 3q27.1-qter fragment (~ 15 Mb) is expected to be within the resolution of karyotyping, it might have been masked as multiple segments were involved in the complex rearrangements and thus were not recognized in the initial karyotyping. Due to the limitations of the OGM technique [29], the short arm of chromosome 22 was not covered by fluorescent markers, and therefore, the rearrangements between chromosome 22 and chromosome 21 could not be well detected. Other methods (such as FISH) may be recommended to further verify the structure of the variant and the location of the chromosome 22 breakpoint. The limitation of our study was that FISH was not conducted due to the lack of site-specific fluorescent probes.

In case 2, the abnormal morphology of chromosomes X and 8 on karyotyping was initially speculated to be due to an insertion event between the two chromosomes. This may be attributed to the difficulty of interpreting various chromosome morphologies and the subjective error on the part of the analyst. Encouragingly, OGM showed a powerful ability to correct these errors and detected multiple rearrangement events containing 7 chromosome breakpoints. The corrected virtual chromosome acceptor breakpoint for the inserted chromosome Xq23q23 was revealed at chromosome 10q26.12; two disconnected deletion segments were identified at chromosomes Xq22.2q23 and Xq23q24, and an inversion event was identified at chromosome 8q21.2q24.3.

In case 3, the OGM assay confirmed the rough karyotyping result, with refinement revealing a more complex rearrangement originating from 9 breakpoints, and provided a molecular characterization of karyotypically apparent simple insertions to demonstrate previously underappreciated complexities.

Additionally, we described a cryptic balanced chromosomal translocation in the father in case 4 that caused unbalanced genomic dosage changes in the fetus. In this case, a cryptic translocation t(2;9)(q37.3;p24.1) was clearly identified by OGM but not by conventional karyotyping. The translocated fragments on chromosome 2 and chromosome 9 are less than 5 Mb in length; therefore, they were undetectable by karyotyping, the routine resolution of which is estimated to be 5 ~ 10 Mb on average. The fetus inherited der(9)t(2;9)(q37.3;p24.1) from the father. The unbalanced genomic changes were expected to contribute to adverse pregnancy outcomes for the fetus and pregnancy. Carriers of cryptic balanced chromosomal translocations are prone to adverse reproductive events, such as infertility, recurrent abortion, suspended embryo development, stillbirth, teratogenesis and birth defects [30–32]. Thus, this kind of aberration, which is characterized by occult findings during routine cytogenetic analyses and serious clinical consequences, needs to be taken seriously. Finer-resolution molecular karyotypes would improve the genetic counseling and clinical treatment strategies for these kinds of cases. Together with previous reports, our findings show that OGM performs highly effectively in identifying cryptic chromosome rearrangements in this study. In a similar clinical scenario, especially in certain countries or labs that cannot easily obtain FISH probes, the OGM assay would be a powerful and accessible tool to detect cryptic translocation carriers, which would be valuable for offering reproductive guidance [8].

The main techniques for detecting structural variations include G-banded karyotyping, CMA, FISH, and NGS, each of which has some well-known advantages and disadvantages. As the most basic analysis of chromosome morphology, karyotyping is capable of detecting most types of balanced and unbalanced SVs as well as numerical chromosomal aberrations, but its resolution is limited to approximately 5–10 Mbp. CMA, which is based on DNA hybridization, offers a resolution of several hundreds to thousands of base pairs in the form of deletions and duplications, but it is not able to detect balanced SVs; FISH can only detect anticipated rearrangements when targeted probes are available; and SKY analysis has limited resolution in delineating cryptic chromosomal rearrangements. Recently, WGS paired with second-generation (i.e., short-read sequencing) or third-generation (i.e., long-read sequencing) technology has been applied to detect SVs. Nevertheless, the relatively short DNA strands used for second-generation sequencing present significant limitations, as long regions of high similarity tend to be difficult or impossible to analyze, whereas high costs and computational challenges currently impede the widespread application of third-generation sequencing. In contrast to these techniques, OGM has extremely high resolution and is capable of comprehensively detecting balanced and unbalanced SVs as small as 30 kb [17], which are unprecedented with karyotyping and CMA. For this reason, OGM has tremendous potential for the discovery of genetic causes of infertility, recurrent spontaneous abortion and histories of adverse pregnancy outcomes, as shown in this and other studies [7, 8, 16].

Despite its high accuracy in resolving all types of chromosomal SVs, OGM has inherent limitations relative to karyotyping in detecting breakpoints lying within large, unmappable repetitive regions, such as centromeres, the p arm of acrocentric chromosomes, or stretches of constitutive heterochromatin. Hence, Robertsonian translocations (RTs) and other whole-arm translocations are considered beyond the current technical capability of OGM and might be prone to false positive and false negative results from this technique [17]. The identification of pathogenic structural variants in these regions should be validated in combination with clinical or other techniques to ensure the authenticity of the results in cases similar to case 1. The limitations of our study are mainly that a sequencing analysis of each rearrangement breakpoint junction was not performed, leaving the mechanisms of their generation elusive in each case, and that, regrettably, FISH was not performed to validate the results, although the rearranged segments were sufficiently large to guarantee the accuracy of the OGM results.

## Conclusions

Overall, due to the hidden nature and uncertainty of SVs, the comprehensive analysis of all SVs requires a combination of OGM with other current techniques. The OGM demonstrated highly effective performance in clinical practice in identifying CCRs and cryptic translocations in clinical practice. Our study confirmed OGM as a solid alternative approach to karyotyping, FISH, and CMA for the detection of chromosomal structural rearrangements, including CCRs and cryptic translocations.

## Data Availability

The datasets used and/or analyzed during the current study are available from the corresponding author on reasonable request.

## Abbreviations

OGM: Optical genome mapping
SVs: Structural variants
CNVs: Copy number variants
CMA: Chromosomal microarray analysis
CCRs: Complex chromosomal rearrangements
FISH: Fluorescence in situ hybridization
SKY: Spectral karyotyping
NGS: Next-generation sequencing
Its: Insertional translocations
GS: Genome sequencing
